# Comparison of Clinical Interventions between Student Pharmacists on Advanced Pharmacy Practice Experiences in Indianapolis, Indiana versus Eldoret, Kenya

**DOI:** 10.3390/pharmacy11030092

**Published:** 2023-05-30

**Authors:** Rakhi Karwa, Monica L. Miller, Ellen Schellhase, Susie Crowe, Imran Manji, Shelby Albertson, Monica Frauhiger, Sonak Pastakia

**Affiliations:** 1 The Academic Model Providing Access to Healthcare, Moi Teaching and Referral Hospital, Eldoret 30100, Kenya; 2 Department of Pharmacy Practice, College of Pharmacy, Purdue University, West Lafayette, IN 47906, USA; 3 Sydney and Lois Eskenazi Hospital, Indianapolis, IN 46202, USA; 4 Carle Foundation Hospital, Urbana, IL 61801, USA

**Keywords:** Kenya, advanced pharmacy practice experience (APPE), clinical interventions, international pharmacy, student interventions

## Abstract

Student pharmacists can have a positive impact on patient care. The objective of this research was to compare clinical interventions made by Purdue University College of Pharmacy (PUCOP) student pharmacists completing internal medicine Advanced Pharmacy Practice Experiences (APPE) in Kenya and the US. A retrospective analysis of interventions made by PUCOP student pharmacists participating in either the 8-week global health APPE at Moi Teaching and Referral Hospital (MTRH-Kenya) or the 4-week adult medicine APPE at the Sydney & Lois Eskenazi Hospital (SLEH-US) was completed. Twenty-nine students (94%) documented interventions from the MTRH-Kenya cohort and 23 (82%) from the SLEH-US cohort. The median number of patients cared for per day was similar between the MTRH-Kenya (6.98 patients per day, interquartile range [IQR] = 5.75 to 8.15) and SLEH-US students (6.47 patients per day, IQR = 5.58 to 7.83). MTRH-Kenya students made a median number of 25.44 interventions per day (IQR = 20.80 to 28.95), while SLEH-US students made 14.77 (IQR = 9.80 to 17.72). The most common interventions were medication reconciliation/t-sheet rewrite and patient chart reviews for MTRH-Kenya and the SLEH-US, respectively. This research highlights how student pharmacists, supported in a well-designed, location-appropriate learning environment, can positively impact patient care.

## 1. Introduction

United States (US) schools of pharmacy have increased the number of international Advanced Pharmacy Practice Experiences (APPE) being offered, many of which occur in low- and middle-income countries (LMICs) [1,2]. These elective experiences are often viewed as observational with minimal impact, which may result from the investments in partner relationships, infrastructure, and education [2,3]. For nearly 20 years, Purdue University College of Pharmacy (PUCOP) has partnered with Moi University and Moi Teaching and Referral Hospital (MTRH) in Eldoret, Kenya [4]. This long-standing collaboration has utilized the five American College of Clinical Pharmacy (ACCP) Global Health Pillars of Engagement to develop and maintain an eight-week international internal medicine APPE [4,5]. There is evidence for students’ positive impact on patient care within high-income countries (HICs) APPEs and within the PUCOP internal medicine APPE in Eldoret, Kenya but limited documentation of student impact in other LMIC settings [6,7,8,9,10,11,12,13,14,15,16]. There is also a lack of documentation comparing student interventions on internal medicine APPEs within each setting. Additional research is critical to documenting the impact of student learning within an international patient care experience, which is categorized as an elective APPE due to geography [17,18]. To address this issue, this brief research report aims to compare and contrast clinical interventions made by PUCOP student pharmacists completing internal medicine APPEs in Kenya and the US. 

## 2. Materials and Methods

The research was conducted as a retrospective analysis of clinical pharmacy interventions made by PUCOP student pharmacists participating in either the eight-week global health APPE at Moi Teaching and Referral Hospital (MTRH-Kenya) or the four-week adult medicine APPE at the Sydney & Lois Eskenazi Hospital (SLEH-US). The SLEH-US APPE was chosen for comparison based on its similarities to the MTRH-Kenya APPE, which included onsite PUCOP faculty precepting, a location within public sector academic medical centers and interdisciplinary inpatient internal medicine services with team-based rounding. In addition, both the Kenyan and US APPEs share core objectives which encompass Center for the Advancement of Pharmacy Education (CAPE) outcomes and incorporate core Entrustable Professional Activities (EPAs) in the following domains: Patient Care Provider, Interprofessional Team Member, Population Health Promoter, Information Master, and Practice Manager [17,19,20]. The outcomes, objectives, and example activities for each site can be seen in Table 1. In addition, a description of each practice site can be seen below. 

MTRH: Kenya’s second largest referral hospital serves a catchment population of over 16 million people and is the main referral center in western Kenya. This teaching hospital hosts learners from many health professional programs from Kenya and the US (such as medicine, nursing, nutrition, and pharmacy). PUCOP students provided care in the adult internal medicine wards with an interprofessional healthcare rounding team daily, supervised by a Kenya-based PUCOP faculty member. Each ward has 48 beds, often shared between two patients, often serving up to 60 patients at any time. Since 2004, this APPE has precepted 315 students.

SLEH: The safety-net, teaching hospital, and level I trauma center within Indianapolis, Indiana, provides care for underserved patients in an urban setting and hosts medical and pharmacy students and residents along with learners from different health professions across the state. PUCOP students round with a faculty member and an interprofessional medical team on one of six adult medicine teaching teams that care for up to 20 patients. Since 2009, this APPE has precepted 160 students. 

Interventions were recorded for PUCOP students who completed APPEs over two years utilizing convenience sampling methodology. Data, including the number and types of accepted interventions, were collected using a standard intervention documentation form (Table S1), previously utilized to capture interventions from PUCOP and Kenyan pharmacy interns at MTRH-Kenya [11]. The form was piloted on the SLEH-US APPE and modified to best meet the needs of both practice sites. The items being documented in this form are activities that summarily meet the core outcomes of a standard internal medicine experience and are routinely used to assess student growth. Standard electronic programs which collect pharmacist interventions, with associated cost-savings, were avoided due to their inability to be applied to interventions in Kenya. Intervention tracking did not document any patient-specific information. MTRH-Kenya students collected data for weeks three through seven, and SLEH-US students collected data during weeks two through four of the APPE to avoid the time period in which preceptors actively attended were made or as soon as possible and tallied by the student at the end of the day. This information (transcribed from paper data collection forms) was forwarded to the preceptors as an electronic spreadsheet and transferred to a password-protected database weekly. 

Descriptive statistics were used to compare and contrast the intervention data made by PUCOP student pharmacists. The primary endpoint was the median number of interventions per patient per student, which was analyzed using a Wilcoxon Rank Sum test, with a *p*-value < 0.05 deemed statistically significant. The secondary endpoints were the most common intervention type recorded in each setting and specific intervention types. The two-sample test of proportions assessed whether statistically significant differences (*p*-value < 0.05) occurred when comparing the intervention type students made in both settings. The study received Institutional Review Board approval at Purdue University. 

## 3. Results

Over two academic years, students completed the MTRH-Kenya (n = 31) and SLEH-US APPEs (n = 28). Twenty-nine students (94%) from the MTRH-Kenya cohort and 23 (82%) from the SLEH-US cohort were included for analysis as a result of having documented interventions for the full-time period. Seven students were not included in the analysis due to missing or incomplete intervention forms. Reasons for missing or incomplete forms included student rotation absence, time away from experience for other educational activities or incomplete data transcription/loss of forms. Table 2 highlights the total number of interventions and patients served for each cohort. While a difference was not seen in the median number of patients served per day, a statistically significant difference was observed in the median number of interventions between the groups, with the MTRH-Kenya students completing more interventions per patient per day. The most common interventions were medication reconciliation/t-sheet rewrite, which required rewriting all currently prescribed treatments, and patient chart reviews for MTRH-Kenya and the SLEH-US, respectively. Notable interventions documented only at MTRH-Kenya were writing intravenous administration instructions and obtaining laboratory and/or clinical monitoring (including blood pressure and blood sugar).

**Figure 1:**
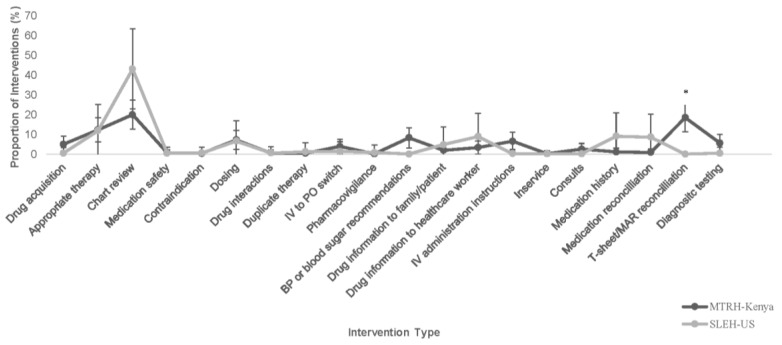
Student Pharmacist Interventions at MTRH-Kenya and SLEH-US by Intervention Type. MTRH = Moi Teaching and Referral Hospital; SLEH-US = Sidney & Lois Eskenazi Hospital-United States; BP = blood pressure; T-sheet = treatment sheet; MAR = medication administration record. + Intervention type ranges represent a 95% confidence interval. * *p* < 0.05.

Figure 1 shows the proportion of student interventions by intervention type. All intervention types were similar between groups except for the statistically significant increase in treatment sheet (t-sheet)/medication administration reconciliation interventions completed by the MTRH-Kenya student pharmacists.

## 4. Discussion

Student pharmacists completing an internal medicine APPE in an LMIC (housed within a faculty-led, locally supported program) were able to provide significantly more clinical interventions when compared to a similar internal medicine APPE in a HIC setting. The study highlighted that student pharmacists cared for a similar number of patients per day between the cohorts, however, the MTRH-Kenya cohort documented higher interventions per patient and interventions per day. Despite similarities in the socioeconomic population served by both hospitals, this finding may suggest that patients have a greater magnitude of need within an LMIC. Additionally, with the exception of medication reconciliation/t-sheet rewrites, few differences in the proportion of intervention types were observed between the cohorts, demonstrating the similarities of these experiences and the opportunities for students in both settings to provide patient care and address educational outcomes. These findings can contribute to the conversation about the educational value of well-developed international APPEs, and the impact of active engagement during an APPE, regardless of the geographic location of the practice site [3].

The difference observed in medication reconciliation/t-sheet rewrites completed during the MTRH-Kenya APPE may be due to the lack of highly protocolized care and services often seen in US hospitals. For example, medication reconciliation programs have been implemented in US hospitals as part of Joint Commission Standards [21]. However, in the MTRH-Kenya APPE setting, these US-focused standards are not in place. Additionally, at SLEH, medication reconciliations are often completed while the patient is still in the emergency department. However, medication reconciliations are not completed when patients are admitted through the casualty (emergency) department at MTRH, leaving this important task to be completed after the patient is admitted to the ward. These differences allowed for a greater opportunity for student pharmacists to aid in patient care through medication reconciliation at MTRH.

As Steeb and colleagues’ study highlighted, students participating in international APPEs, particularly in LMICs, build knowledge, skills, and attitudes that meet ACPE accreditation standards [17,22]. The quantity and variety of interventions completed by the MTRH-Kenya cohort highlight the transferrable skills that student pharmacists participating in this APPE could obtain during these elective APPEs [18,23,24]. Based on the intervention types, it is evident that students learned patient care skills, including professional communication, interprofessional collaboration, and clinical decision-making, which are all necessary according to ACPE accreditation standards [19,25]. As healthcare education and training moves to the utilization of EPAs, the documented patient care skills demonstrated in this study address pharmacy and global health EPAs [20,26,27]. 

Student success, defined as the student’s ability to make clinical interventions and integrate with a Kenyan medical team, maybe largely due to the intentional design of the MTRH-Kenya APPE, which aligns with the ACCP Global Health Pillars of Engagement [3]. PUCOP and their local Kenyan partners have a long-standing, committed partnership, lasting almost 20 years [28,29,30]. To aid in the sustainability of this learning environment, PUCOP and Kenyan leadership have partnered around a common vision for learning. Additionally, the MTRH-Kenya APPE partners PUCOP student pharmacists with Kenyan learners to increase reciprocal learning opportunities. A 2011 study at MTRH observed that Kenyan pharmacy interns completed more interventions than their PUCOP student counterparts [11]. Partnering PUCOP student pharmacists with Kenyan learners increases cultural context and enhances their ability to connect with patients. 

Student success is also a product of the long-standing PUCOP investment in developing Kenyan leaders. This investment has increased pharmacy’s presence within the MTRH system and improved patient care services [4,31,32,33]. Kenyan providers are supportive of pharmacy learners in the adult medicine wards and request their presence. PUCOP’s investment has enriched the experience for patients and learners alike. The placement and integration of the PUCOP faculty as part of the on-the-ground team at MTRH have also contributed to student success. By having integrated faculty, Kenyan pharmacists are provided learners to support their care programs and assist in managing daily needs. The faculty focuses on managing student learning, allowing Kenyan pharmacists to prioritize the optimization of patient care. A final key to student pharmacist success is that all PUCOP students complete a preparatory course that aims to ensure students are aware of the healthcare institution and institutional practices to decrease their acclimation period allowing for expedited integration into the team [34]. When global health programs utilize these strategies and the ACCP Global Health Pillars of Engagement as guidance in developing student engagement opportunities, it can help support the ultimate goal of improving the overall health and well-being of the local community while also creating a robust clinical learning environment. 

This research had several noteworthy limitations. First, the APPE sites were chosen for their similarities as described in the methods. However, there were some key differences in duration and students completing the APPE. To minimize the impact on the comparison, data analysis described interventions per patient per day. It is still possible that students made more interventions in the MTRH-Kenya APPE because they spent more time on rotation which may have translated to improved familiarity with the setting and an increased number of interventions. Students participating in the SLEH-US APPE were randomly assigned to the APPE, while in contrast, MTRH-Kenya students elected the experience and completed an application for a limited number of positions. It is possible that the selection process for the MTRH-Kenya APPE selected more engaged students who chose to participate in the APPE. The documentation form utilized, adapted from the form used in the 2011 study, was created for use in the MTRH-Kenya setting and was subsequently applied to the SLEH-US setting. To minimize this limitation, the form was piloted prior to its use to address any needed changes or additions to the form. Additionally, student pharmacists only documented accepted interventions and did not capture the total number of interventions provided to the medical teams in an effort to reduce the documentation burden and allow a focus on patient care. 

## 5. Conclusions

This brief research report demonstrates that student pharmacists rounding with interprofessional medical teams in Kenya and the US can complete similar interventions per day. Through this comparison, the MTRH-Kenya students clearly demonstrated the development of transferrable professional skills. Finally, this research highlights how student pharmacists, supported in a well-designed locally appropriate learning environment, can positively impact patient care. More studies are needed to determine correlations between US and international APPEs to determine how wide stretching these correlations are in a variety of different settings. 

## Figures and Tables

**Table 1:** Sample core outcomes, objectives, and activities of internal medicine rotations in Kenya and the United States.

Shared APPE Outcomes and Objectives	MTRH-Kenya Activity	SLEH-US Activity	EPA Domain ^a^	CAPE Outcome ^b^
**Exemplify Clinical Competence:** Demonstrate appropriate clinical knowledge Design patient-specific pharmacotherapy plans Implement specific and measurable monitoring plans Demonstrate a questioning mindset through daily self-directed identification of clinical questions Identify and advocate for preventative screenings and care	Interdisciplinary rounds Patient counseling Screening for HIV and TB Identification of literature for patient care Daily medication administration record review	Interdisciplinary rounds Patient counseling Screening for immunization needs Identification of literature for patient care Admission medication reconciliation	a, c, d, f	1.1, 2.1–2.4, 3.1–3.5, 4.2
**Effective Communication Skills:** Develop appropriate professional relationships and communication with members of the health care team, preceptor(s), and students Advise healthcare professionals on appropriate drug therapy based on efficacy, safety, and cost-effective prescribing Deliver informative and appropriate presentations Provide patient education using appropriate language and assessment strategies	Interdisciplinary care rounds Topic discussions Patient presentations Journal Club	Interdisciplinary care rounds Topic discussions Patient presentations Journal Club	a, b, d	3.2, 3.4, 3.6, 4.2, 4.4
**Gain Institutional Pharmacy Awareness:** Develop an understanding of pharmacy policies and procedures Participate in the identification of drug errors and adverse drug reactions	Participation in centralized pharmacy activities Identify drug errors and report adverse drug reactions	Orientation to pharmacy workflow Utilize formulary options	c, e	2.2
**Professionalism:** Exhibit the characteristics of a professional Demonstrate empathy in interactions with patients and the healthcare team Practice time management skills	Interdisciplinary rounds Patient counseling Self-evaluations Interviewing workshop Weekly cultural debriefs	Interdisciplinary rounds Patient counseling Self-evaluations	a, b, e, f	4.1, 4.4

^a^ EPA domain: a = patient care provider; b = interprofessional team member; c = population health promoter: d = information master; e = practice manager; f = self-developer [20]; ^b^ CAPE outcome domain: 1 = foundational knowledge; 2 = essentials for practice and care; 3 = approach to practice and care; 4 = personal and professional development [19]; MTRH = Moi Teaching and Referral Hospital; SLEH-US = Sidney & Lois Eskenazi Health-United States; EPA = Entrustable Professional Activities; CAPE = Center for the Advancement of Pharmacy Education; HIV = Human Immunodeficiency Virus; TB = Tuberculosis.

**Table 2:** Comparison of Student Pharmacist Interventions.

	Kenya-MTRH Student Pharmacists N = 29	US-SLEH Student Pharmacists N = 23	*p* Value
Total number of interventions	14,950	3529	n/a
Total number of patients	4155	1865	n/a
Patients per day ^a^	6.98 (5.75–8.15)	6.47 (5.58–7.83)	0.92
Interventions per patient ^a^	3.75 (2.96–4.61)	2.40 (1.42–2.80)	<0.01
Interventions per day ^a^	25.44 (20.80–28.95)	14.77 (9.80–17.72)	<0.01

MTRH = Moi Teaching and Referral Hospital; US-SLEH = United States-Sidney & Lois Eskenazi Hospital; IQR = interquartile range; ^a^ Data represented as median with interquartile range.

## Data Availability

The data presented in this study are available on request from the corresponding author.

## Supplementary Materials

The following supporting information can be downloaded at: https://www.mdpi.com/article/10.3390/pharmacy11030092/s1, Table S1: Student Interventions Collection Form.
