# Supplementary material for: Comparison of Clinical Interventions between Student Pharmacists on Advanced Pharmacy Practice Experiences in Indianapolis, Indiana versus Eldoret, Kenya
Source: Pharmacy (Basel). 2023 May 30;11(3):92. doi: 10.3390/pharmacy11030092 (PMC10304254; doi:10.3390/pharmacy11030092)
Supplement: Supplementary file 1 [file pharmacy-11-00092-s001.zip › pharmacy-2276481-supplementary.pdf]

Table S1. **Student Interventions Collection Form** (Student Intervention Data Collection Tool)

Site Name:____ Student Name:____

For the table below only include accepted recommendations

Date	BP/BS monitoring instructions	Drug info to family or patient	Drug info to healthcare worker	IV administration instructions	Inservice	Consults (see code below)	Medication History	Medication reconciliation	T-sheet or MAR reconciliation		Diagnostic and lab testing	Total number of patients
									Rewrite	Other		

BP/BS=Blood pressure/blood sugar; Consults: A = Anticoagulation; H = HIV; T = Tuberculosis; N = Nutrition; E = Endocrine/Diabetes

Site Name:____ Student Name:____

For the table below only include accepted recommendations

Date	Drug Acquisition	Appropriate therapy (drug selection, addition, change)	Chart Review	Medication Safety Issue	Contraindication	Dosing	Drug interaction	Duplicate therapy	IV to PO	Pharmaco-vigilance	Total # of consultations

Adapted from: Pastakia SD, Vincent WR 3rd, Manji I, Kamau E, Schellhase EM. Clinical pharmacy consultations provided by American and Kenyan pharmacy students during an acute care advanced pharmacy practice experience. *Am J Pharm Educ*. 2011;75(3):42.
Document Activities that address domains 2 & 3 of the Center for Advancement of Pharmacy Education 2013 Educational Outcomes (*Am J Pharm Educ*. 2013;77(8):162)
